# Enamel Renal Syndrome: A Case Report

**DOI:** 10.7759/cureus.72359

**Published:** 2024-10-25

**Authors:** Nairica Rebello, Anita E Spadigam, Anita Dhupar, Jochima E Cota

**Affiliations:** 1 Oral and Maxillofacial Pathology, Microbiology and Forensic Odontology, Goa Dental College and Hospital, Panjim, IND

**Keywords:** amelogenesis imperfecta, case report, enamel hypoplasia, gingival fibromatosis, nephrocalcinosis

## Abstract

Amelogenesis imperfecta is a genetic disorder that manifests as a defect in the quality or quantity of enamel in both deciduous and permanent teeth, affecting some teeth or the entire dentition. A fraction of cases occur in conjunction with systemic involvement. Enamel renal syndrome is a rare entity diagnosed based on a combined presentation of amelogenesis imperfecta and renal calculi. The ambiguity surrounding succinct diagnostic criteria results in a significant underestimation of prevalence. A 12-year-old male presented with discoloration of teeth for two years with no relevant past medical or dental history. Intraoral examination revealed yellowish discoloration of the entire dentition and fibrotic gingival enlargement. Abdominal ultrasound revealed renal calculi. Based on the clinical, pathological, and radiographic features, a diagnosis of enamel renal syndrome was made. This case underscores the need for a comprehensive diagnostic workup to prevent complications that may arise from systemic involvement.

## Introduction

Enamel renal syndrome (ERS) was first described by MacGibbon in close relatives of a family [1]. It is described as an uncommon autosomal recessive disorder characterized by hypoplastic amelogenesis imperfecta (AI), delayed tooth eruption, intrapulpal calcifications, gingival enlargement, and nephrocalcinosis [1]. Globally, it has been noted to affect less than one in 100,000 individuals [1]. The diagnosis of ERS is primarily clinical, relying on oro-dental changes as well as renal findings [2]. However, the rarity of this condition coupled with variations in phenotypic presentation renders the diagnosis enigmatic [3]. We present a case of ERS in a male child with no apparent medical history.

Written consent for publication of this case was obtained from the patient's mother as the patient is a minor.

## Case presentation

**Figure 1:**
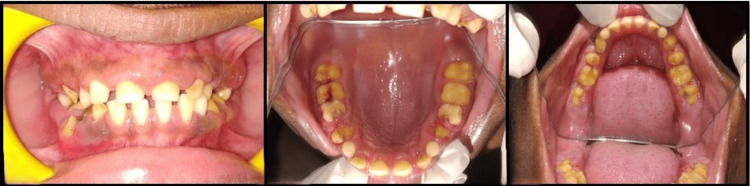
Intraoral photograph showing yellowish discoloration of teeth and generalized grade II gingival enlargement.

A 12-year-old male presented to a tertiary care dental hospital with the complaint of discoloration of teeth for two years. The past medical and dental histories were unremarkable. Family history included a consanguineous marriage between the parents and no history of similar findings in any closely related member of the family. Physical examination revealed a moderately built child of average height and weight. Intraoral examination revealed yellowish discoloration involving the entire mixed dentition and generalized grade II gingival enlargement. On palpation, the teeth had an irregular and flaky surface. The gingiva was firm and fibrotic and bled on probing (Figure 1).

**Figure 2:**
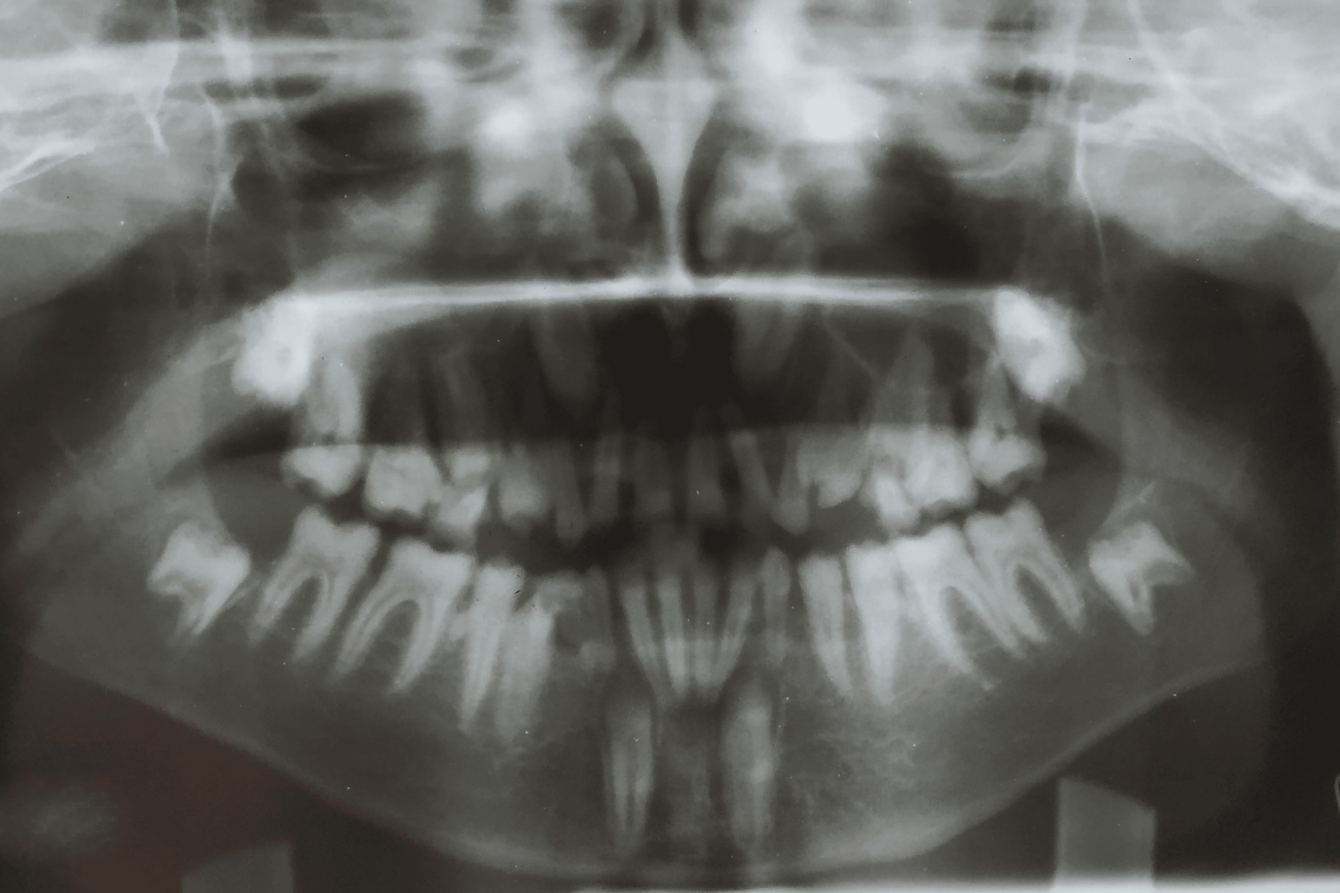
Orthopantomogram showing inadequate enamel thickness on all teeth.

No significant increase in pocket depth was noted. Orthopantomography revealed inadequate enamel in all the teeth (Figure 2).

**Figure 3:**
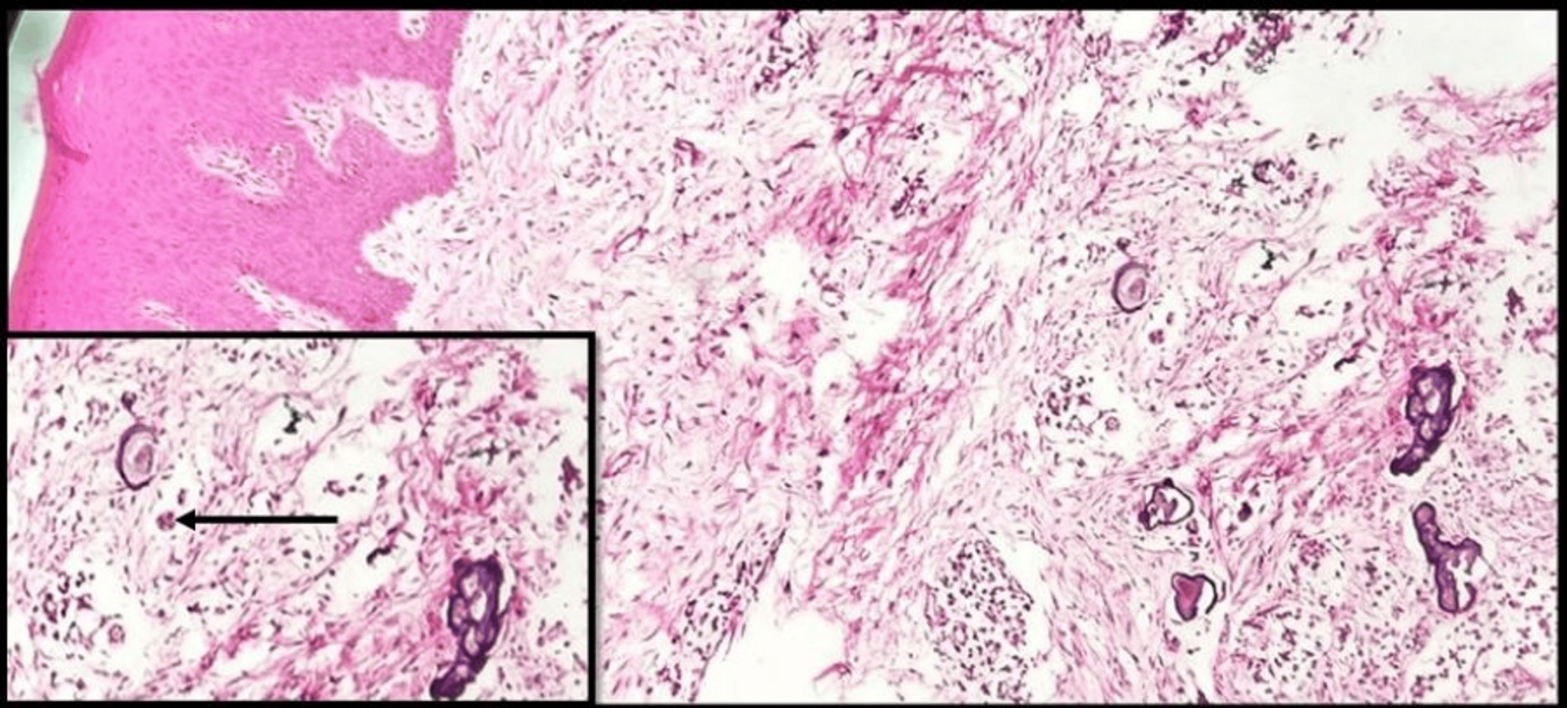
Photomicrograph of hematoxylin and eosin-stained section showing calcifications (10x) and an arrow pointing to an odontogenic cell rest (inset 40x).

Gingivectomy was performed, and hyperplastic gingival tissue was processed for histopathological evaluation. The hematoxylin and eosin-stained section revealed stratified squamous parakeratinized epithelium overlying a fibro-cellular connective tissue stroma consisting of collagen fiber bundles, fibroblasts, blood capillaries, and focal chronic inflammatory infiltrate. Deeper stroma showed the presence of dystrophic calcifications, which appeared laminated, in close proximity to odontogenic cell rests (Figure 3).

Renal ultrasound revealed bilateral renal calculi. However, the results of laboratory investigations including renal function tests, serum calcium, parathormone, and vitamin D were normal. The *FAM20A* gene mutation could not be assessed due to financial constraints.

Based on the above features, a diagnosis of ERS was made. The patient was referred to a nephrologist and advised regular dental and medical follow-up.

## Discussion

**Figure 4:**
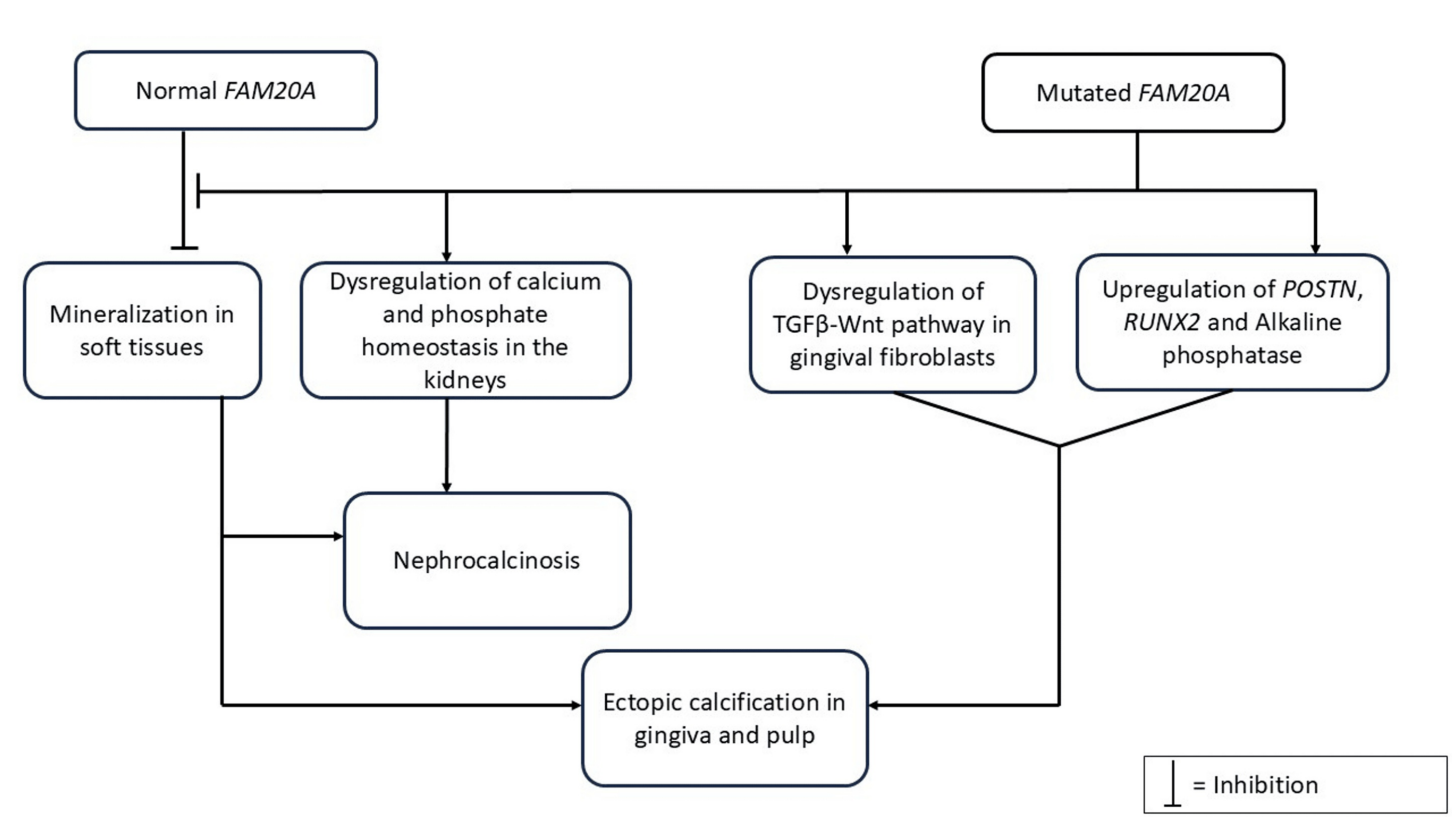
Schematic representation of proposed pathogenesis of enamel renal syndrome. POSTN: periostin; RUNX: runt-related transcription factors; TGF: transforming growth factor; Wnt: wingless/integrated Image credits: Nairica Eurico Rebello

Enamel renal syndrome is hypothesized to be associated with mutations in the *FAM20A* gene, which results in aberrant mineralization of the gingiva, dental follicles, kidneys, and lungs (Figure 4) [4,5,6].

A hereditary association has been suggested based on the finding of consanguinity in several cases, including the present case [5].

Phenotypic variation results in a spectrum of presentations with AI and nephrocalcinosis common to all [2]. Muriel de la Dure‑Molla et al. [2] proposed a list of diagnostic criteria for patients with ERS, of which microdontia, generalized spacing, flat occlusal surfaces of molar crowns, and yellow discoloration of teeth were present in the current case. 

A combination of clinico-radiographic findings and histopathological evidence is crucial to arrive at a diagnosis. Among the radiographic features reported, our patient exhibited a generalized reduction in enamel thickness.

Histopathological sections revealed ectopic mineralization close to odontogenic rests similar to that reported by Feller L et al. [7]. The calcifications resembled psammomatous calcifications similar to those reported by Wang et al. [8,9,10].

## Conclusions

Dentists play a crucial role in the diagnosis of ERS, particularly in the absence of any renal findings. The presence of AI and gingival enlargement even in the apparent absence of any other symptoms is sufficient to warrant further systemic investigation. Timely diagnosis and multidisciplinary management aid in minimizing sequelae associated with renal involvement. 
